# Health Effects from Swimming Training in Chlorinated Pools and the Corresponding Metabolic Stress Pathways

**DOI:** 10.1371/journal.pone.0119241

**Published:** 2015-03-05

**Authors:** Jiang-Hua Li, Zhi-Hui Wang, Xiao-Juan Zhu, Zhao-Hui Deng, Can-Xin Cai, Li-Qiang Qiu, Wei Chen, Ya-Jun Lin

**Affiliations:** 1 Key Laboratory of Training Monitoring and Intervention on the Sports in Water, State Sports General Administration, Institute of Physical Education, Jiangxi Normal University, Nanchang, China; 2 Jiangxi Provincial Key Laboratory of Chemical Biology, Jiangxi Normal University, Nanchang, China; 3 Beijing Economic Management School, Beijing, China; The University of Iowa, UNITED STATES

## Abstract

Chlorination is the most popular method for disinfecting swimming pool water; however, although pathogens are being killed, many toxic compounds, called disinfection by-products (DBPs), are formed. Numerous epidemiological publications have associated the chlorination of pools with dysfunctions of the respiratory system and with some other diseases. However, the findings concerning these associations are not always consistent and have not been confirmed by toxicological studies. Therefore, the health effects from swimming in chlorinated pools and the corresponding stress reactions in organisms are unclear. In this study, we show that although the growth and behaviors of experimental rats were not affected, their health, training effects and metabolic profiles were significantly affected by a 12-week swimming training program in chlorinated water identical to that of public pools. Interestingly, the eyes and skin are the organs that are more directly affected than the lungs by the irritants in chlorinated water; instead of chlorination, training intensity, training frequency and choking on water may be the primary factors for lung damage induced by swimming. Among the five major organs (the heart, liver, spleen, lungs and kidneys), the liver is the most likely target of DBPs. Through metabolomics analysis, the corresponding metabolic stress pathways and a defensive system focusing on taurine were presented, based on which the corresponding countermeasures can be developed for swimming athletes and for others who spend a lot of time in chlorinated swimming pools.

## Introduction

Chlorination is the most popular method for disinfecting swimming pool water. However, although pathogens are being killed, many toxic compounds, called disinfection by-products (DBPs), are formed. Numerous publications have indicated that DBPs exposure may be related to several diseases [1–3], and Thomas Lachocki, the head of the National Swimming Pool Foundation of USA, has emphasized that the health benefits from swimming must be weighed against the risks of chemical exposure [4]. The epidemiological evidence for adverse health effects from swimming in chlorinated water primarily originate from studies concerning respiratory function and asthma, althoughVillanueva et al. reported a significant increased risk of bladder cancer for swimmers compared with nonswimmers [5]. The chlorination of pools has been associated with an increase in lung epithelium permeability [6], a risk of developing asthma [7], and with respiratory complaints [8]. Typically, trihalomethanes and trichloramines are blamed [4]. However, the findings regarding the association of chlorination with illness are not always consistent. Font-Ribera et al. reported that swimming did not increase the risk of asthma or allergic symptoms in British children [9] but was associated with slightly less respiratory tract symptoms [10], increased lung function and with a lower risk of asthma symptoms, particularly among children with preexisting respiratory conditions [9]. A meta-analysis performed by Goodman et al. demonstrated that the association between asthma and swimming could only be confirmed among competitive swimmers and could not be confirmed among non-competitive swimmers [11]. Extremely few toxicological studies have been performed in the area of swimming exposure and health thus far. Therefore, the health effects from swimming in chlorinated pools and the corresponding stress reactions occurring in our bodies are unclear. Generally, competitive swimmers are the most possible victims of DBPs exposure, because they have to do a lot of high intensive training in swimming pools for years. To reveal the health effects of DBPs exposure from swimming training, the experimental animals were trained in chlorinated water as competitive swimmers for twelve weeks in this study (according to the lifespan of the animals, twelve weeks for rats almost equals ten years for human being, which is a nessary period for an athlete to get a best performance). Their behaviors and appearances were observed during the training program, and then histopathological and metabolomic approaches were used to analyze the health effects and corresponding metabolic stress pathways.

## Materials and Methods

### Animals

Animal welfare and experimental procedures were performed in accordance with the Guide for the Care and Use of Laboratory Animals (Ministry of Science and Technology of China, 2006) and were approved by the animal ethics committee of Jiangxi Normal University. Twenty-four Sprague-Dawley rats, which were three weeks old and weighed 207.1 ± 43.9 g, were commercially obtained from the Department of Laboratory Animal Science, Nanchang University, China. Throughout the study periods, all rats were housed in 590×380×200 mm plastic cages under the following conditions: 20–24°C room temperature, natural light, standard food and free water.

### Treatment

After acclimatization for one week, the 24 rats were randomly distributed into a control group (CG, n = 6) and an experimental group (EG, n = 18), and then a 12-week swimming training program was performed for both groups. Unfortunately, one of the rats accidentally drowned during swimming training; therefore, the final animal number of the EG was 17. The water for the EG was purified using a water purifier and then disinfected using calcium hypochlorite, similar to public swimming pools, whereas the water for the CG was only purified, not chlorinated. Free chlorine in the swimming water was monitored using the N, N-diethyl-p-phenylenediamine (DPD) method. The level of free chlorine in the water for the EG was adjusted to 1.4–1.6 mg/L before swimming training (the ideal level recommended by the World Health Organization for public pools [12]); no free chlorine was detected in the water for the CG. The training was performed once a day, five days a week. When training, a screw nut approximately 3% of their mean body weight was tied to the top end of the tail of each rat, and all rats were kept in the special pools with water of 60 cm depth (water temperature 25–30°C, pH 6.5–7.0) until fatigued (submerged below the surface for five seconds twice). The fatigued rats ceased training immediately, were removed from the water for a short break, showered with running water and then dried with hair dryers.

### Chlorination DBP measurements

Two typical classes of chlorination DBPs, chloroform and chloramines, were measured at the 20th minute of every training session. Chloroform was measured using a gas chromatograph (SHIMADZU GC-2010) with a HP-5 chromatographic column (30 m×0.32 mm×0.25 μm) in accordance with the Chinese standard test methods for organic substances in drinking water (GB/T 5750.8–2006). Chloramines were measured using a colorimeter (HKM II, Guangdong Huankai Microbial Sci. & Tech. Co. Ltd.) based on the DPD method.

### Behaviors and appearances

Behaviors and appearances of the rats were observed throughout the study. Body weights were measured using an electronic balance.

### Swimming capacity test

After the 12-week swimming training and one day of rest, a swimming capacity test was performed. The duration from entry into water until the exhaustion of each rat was recorded. The pool water used in the test was running water supplied by the municipal water company. To increase the intensity and to shorten the time, a 13.7 g screw nut was tied to the top end of the tail of each rat in the test. The exhaustion criterion was set such that rats remained submerged below the surface for ten seconds [13].

### Urine samples

The day before the swimming capacity test, 24-hour urine samples were collected with metabolic cages (NaN_3_ preservation) and then centrifuged at 3000 r/min at 4°C for 10 min. Approximately 4 ml supernatant aliquots were transferred into 5 ml Eppendorf tubes and stored at −80°C for nuclear magnetic resonance (NMR) testing.

### Histopathology

The day after the swimming capacity test, animals were sacrificed with an overdose administration of pentobarbiturate (120 mg/kg). First, a gross anatomy dissection was performed on the animals, and tissues of their five major organs, i.e., heart, liver, spleen, lungs and kidneys, were collected and fixed with 4% formaldehydum polymerisatum. Twelve hours later, the fixed tissues were embedded in paraffin for sectioning, and then a regular histopathological analysis was performed using hematoxylin and eosin staining and optical microscopes.

### NMR measurements

Urine samples were thawed at room temperature, and then 400 μL urine was mixed with 200 μL phosphate buffer (pH 7.4,0.2 M NaH_2_PO_4_/Na_2_HPO_4_), with 10% D_2_O as a field lock and with 0.05% sodium 3-trimethylsilyl-(2,2,3,3-2H4)-1-propionate (TSP) as a chemical shift reference. After centrifugation at 13000 g for 10 min, the supernatants were transferred into 5 mm NMR tubes and measured using a standard one dimensional ^1^H pulse sequence with water suppression (Noesypresat) on a Bruker DRX400 spectrometer operating at 400.13 MHz ^1^H resonance frequency and at 298 K.

### Data processing

All ^1^H NMR Spectra were manually corrected for phase and baseline distortions and referenced to the TSP signal at 0 ppm using Top Spin software version 3.0 (Bruker Biospin, Germany). Integration was performed over a 10.00–0.02 ppm region, with a bucket width of 0.02 ppm. Regions corresponding to the spectrum signals of water and of urea (6.20–4.20 ppm) were excluded, and the integration of each region was normalized to the sum of the total spectrum to obtain the urine metabolite data (S1 Table).

### Statistical analysis

Principal component analysis (PCA) was conducted on the urine metabolite data for pattern recognition using SIMCA-P+ software version 10.0 (Umetrics, Umea, Sweden). Before PCA, data were subjected to orthogonal signal correction (OSC) and unit variance scaling. Other statistical analyses were performed using IBM SPSS Statistics software version 20.0 (SPSS Inc., Chicago, IL, USA). The durations of the animals in swimming capacity test were expressed as the mean value ± standard deviation (SD) and compared using independent t-tests. The positive rates in histopathological analysis were evaluated by Chi-square (χ2) test. The significance level was set at 0.05.

## Results and Discussion

### Concentrations of the typical chlorination DBPs

Concentrations of the two typical classes of chlorination DBPs (chloroform and chloramines), which were measured at the 20^th^ minute of the swimming training, were 0.7±0.05 μg/L and 1.05±0.12 mg/L in the water for the EG, and none of these chemicals were detected in the water for the CG. The 20^th^ minute was approximately the middle point of the training session (the duration of each training session was approximately 40 minutes); thus, the concentrations of the DBPs at this point were selected to represent the exposure doses. The exposure doses of these two typical classes of DBPs for the EG rats were significantly higher than those for the CG rats, and this experiment was a typical chronic low-dose exposure experiment.

### Behaviors and Appearances

The final body weights of the rats measured before euthanasia were extremely close between the EG and the CG (344.34 ± 34.95 g vs. 337.07 ± 46.00 g, p>0.05). No significant behavior differences were observed between the two groups during the entire experimental period; however, some unusual appearance changes appeared in the EG rats. First, the skin around their eyes became increasingly red with the development of the experiment, and in the ending period of the experiment, bloodstains could be observed in the rims of most rats’ eyes. Second, from the third experimental week on, an increasing number of rats had bloodstains appearing at the tips of their noses; however, approximately two weeks later, this symptom gradually disappeared. Third, their fur became increasingly dry and lackluster, and significant signs of hair loss were observed during the last month.

These results indicated that the fur, respiratory tracts and eyes of the EG rats were severely affected by chlorinated water, although their growth was essentially unaffected. According to our observations, the daily behaviors and sizes of the EG rats were normal, and their final body weights were even slightly heavier than those weights of the control group. Nevertheless, dried and lackluster fur, hair removal, bloody noses and eyes did occur in the EG rats and not to the CG rats. In fact, similar symptoms, red and swollen eyes, dried skin and nasal mucosal congestion, always appear after humans swim in a chlorinated pool; however, the long-term (12 weeks) and high-frequency (5 days a week) of the experimental swimming training caused even worse symptoms in these experimental rats.

Additionally, an interesting phenomenon was observed by comparing the development of the bloody noses and bloody eyes. The bloody noses commonly appeared in the third and in the fourth week; however, approximately two weeks later, this symptom gradually disappeared. The significantly bloody eyes commonly appeared in the ending period of the experiment; however, this problem was becoming worse during the study, and no signs of improvement appeared. The bloody noses appeared first, suggesting that respiratory tracts may be more vulnerable to the irritants from the chlorinated water than eyes; the gradually disappearing symptom suggests that respiratory tracts may have some adaptability to chlorinated water possibly because of the protection from nasal mucous. In contrast, without the mucosal protection, the bloody eyes were becoming increasingly significant during the entire experiment, although this symptom appeared later than the bloody noses.

Therefore, the eyes and skin may be the organs that must be the focus of concern regarding permanent damage induced by irritants from chlorinated water, rather than respiratory tracts, although respiratory symptoms were the most emphasized toxic risk of swimming exposure in recent decades.

### Swimming capacity test

The duration period from the entry into water until the rats reached exhaustion was significantly shorter for the EG rats compared with the CG rats (29.74±11.50 vs. 39.15±9.85 minutes, p<0.05), indicating that the training effects were significantly impaired by the chlorinated water.

### Histopathological analysis

The results of histopathological analysis is shown in Table 1. The color and size of the five major organs, the heart, liver, spleen, lungs and kidneys, of all rats were normal, and no significant exudation, hyperplasia, edema, atrophy and other disease symptoms were observed during the gross anatomy dissection. Under light microscopy, no disease symptoms were found in the heart, spleen and kidney sections; however, some disease symptoms were observed in the liver and lung sections.

**Table 1:** The results of histopathological analysis.

Group	N	Gross anatomy		Heart		Liver		Spleen		Lung		Kidney	
		Nor	Pos	Nor	Pos	Nor	Pos	Nor	Pos	Nor	Pos	Nor	Pos
CG	6	6	0	6	0	6	0	6	0	0	6	6	0
EG	17	17	0	17	0	14	3	17	0	0	17	17	0

Note: CG = control group, EG = experiment group, N = number, Nor = normal, Pos = positive.

**Fig 1:**
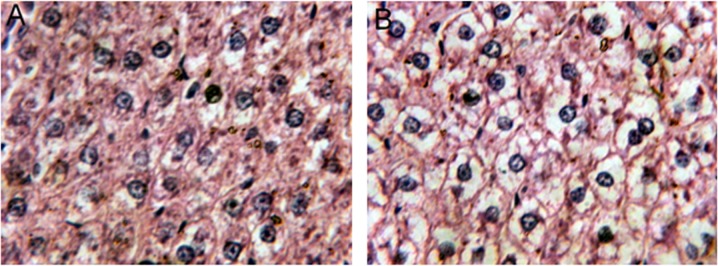
Representative hematoxylin and eosin staining (H&E, 400×) of liver sections. (A) from the control group, (B) from the experimental group. Significant signs of cytoplasm rarefaction and ballooning degeneration were found in the rats from the experimental group, whereas the control group rats had primarily normal cell shapes.

Significant signs of cytoplasm rarefaction and ballooning degeneration were found in the liver sections of 3 rats from the EG (Fig. 1A), whereas the CG rats had primarily intact nuclei and normal cell shapes (Fig. 1B). Three rats from the EG were found liver damage, indicating that the positive rate reached 18 percent. Although this ratio did not show statistical significance when compared with the CG, it still suggests that the liver is most likely an important target organ of DBPs. Because of its unique metabolism and relation to the gastrointestinal tract, the liver is an important target of the toxicity of drugs, xenobiotics, and of oxidative stress [14]. Numerous reports have demonstrated the hepatoxicity of chloroform, which is the most common toxic DBP in chlorinated swimming pools. For instance, Tumasonis et al. reported that a significantly increased incidence of hepatic neoplastic nodules in female rats and a significantly increased incidence of hepatic adenofibrosis in both male and female rats were induced by the chronic ingestion of chloroform [15]. Additionally, Reuber claimed to have found a statistically significant number of hepatic cholangiocarcinomas in female rats with chronic chloroform administration [16].

**Fig 2:**
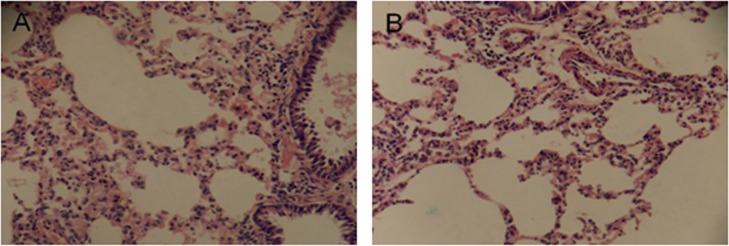
Representative hematoxylin and eosin staining (H&E, 100×) of lung sections. (A) from the control group, (B) from the experimental group. Dilatation and congestion of the capillary vessels around the alveoli, edema fluid in the alveoli, and lymphadenosis under bronchial mucosae were commonly observed in the lung sections from both groups. No significant differences were found between the experimental group and the control group.

Dilatation and congestion of the capillary vessels around the alveoli, edema fluid in the alveoli, and lymphadenosis under the bronchial mucosae were commonly observed in the lung sections from both groups (Fig. 2). No significant differences were found between the EG and the CG.

Compared with liver damage, lung damage was much more commonly detected under microscopes. However, notably, both the rats from the EG and from the CG had almost identical symptoms in their lungs. This phenomenon, combined with the disappearing bloody noses discussed above, suggests that chlorination disinfection may not be the primary factor for lung damage during swimming because the alveoli and bronchi are also protected by attached mucosal-like respiratory tracts. In contrast, we believe that the training intensity, training frequency and water choking might be the primary factors for the observed lung damage. In this study, both groups of rats were trained similar to competitive swimmers, with high frequency and high intensity, which made them inevitably choke much water and become more vulnerable to lung damage. Similarly, studies of elite swimmers in the United States, Canada, Great Britain, Australia, Finland, and in Ireland confirmed that asthma is more common only among competitive swimmers and was not common among non-competitive swimmers^11^. We suppose that the primary differences between the competitive swimmers and non-competitive swimmers are training intensity and frequency, not the pool water.

### Metabolomics

#### Principal component analysis (PCA)

**Fig 3:**
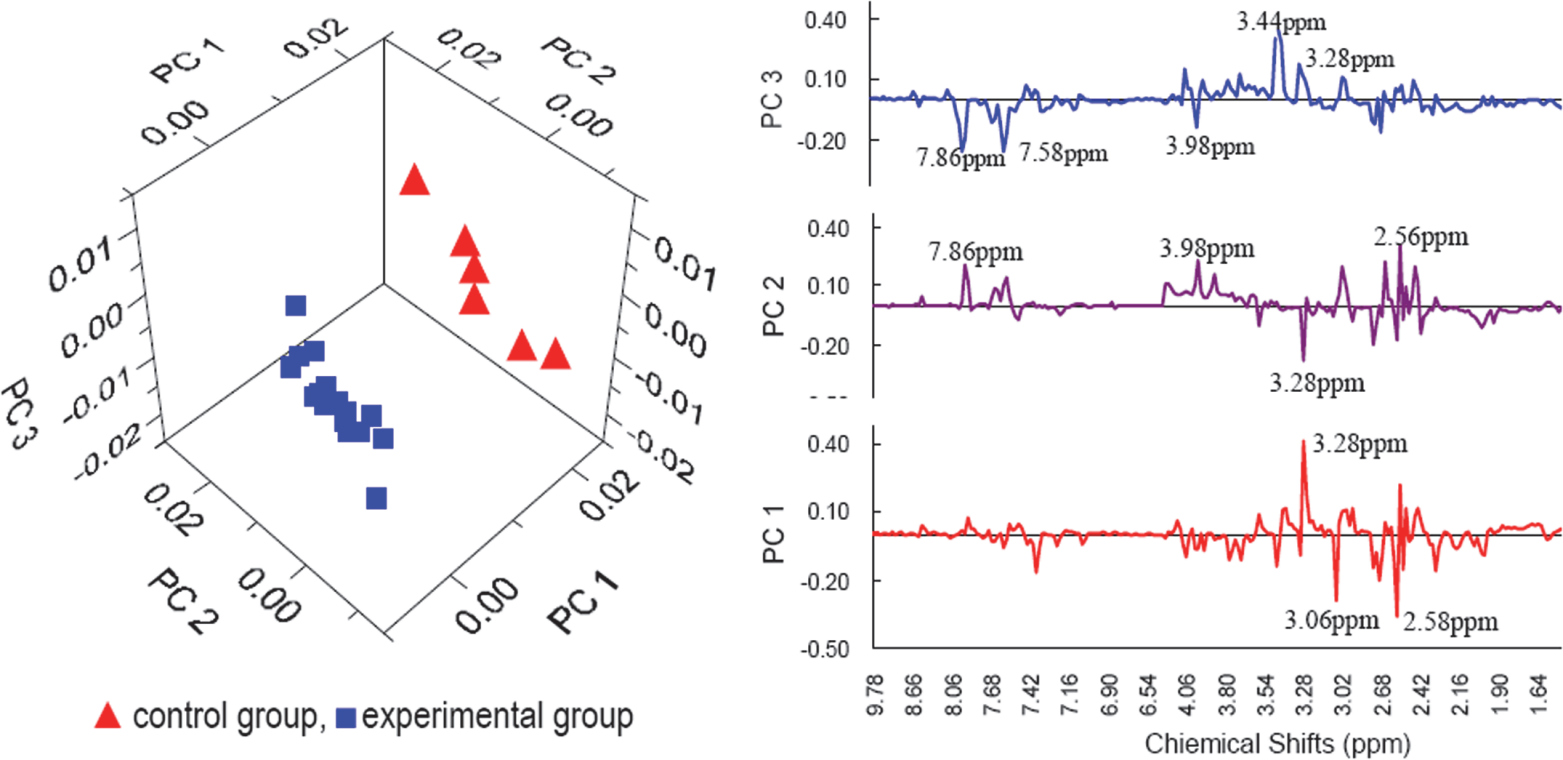
Score plot (left) and loading plot (right) of the principal component analysis. All samples are clustered clearly and correctly into two different groups in the principal component analysis score plot, which suggests that the 12-week swimming training in chlorinated water induced a significant change in the metabolic profiles of the experimental group rats. To identify the primary metabolites that changed under the conditions of this experiment, the chemical shifts in the peaks with large loadings in the first three principal components are labeled in the loading plot.

The first three principal components (PCs) explain 57.3% of the total variance of the raw data and are sufficient to present the differences between the EG and the CG. All samples are clearly and correctly clustered into two different groups in the PCA score plot (Fig. 3), which suggests that the 12-week swimming training in chlorinated water induced a significant change in the metabolic profiles of the EG rats. To identify the primary metabolites that changed under the conditions of this experiment, the chemical shifts in the peaks with large loadings in the first three PCs are marked in the PCA loading plot (Fig. 3). In Fig. 3, we can see that the majority of the differences between the EG and the CG are explained by PC 1 and PC 3. The T tests further confirm that the differences between the groups are significant on PC 1 and PC 3 (p<0.05), not significant on PC 2 (p>0.05). Thereby, the interpretation of PCs in the following parts will focus on PC 1 and PC 3.

#### Peak assignment and changing trends of the assigned metabolites

**Fig 4:**
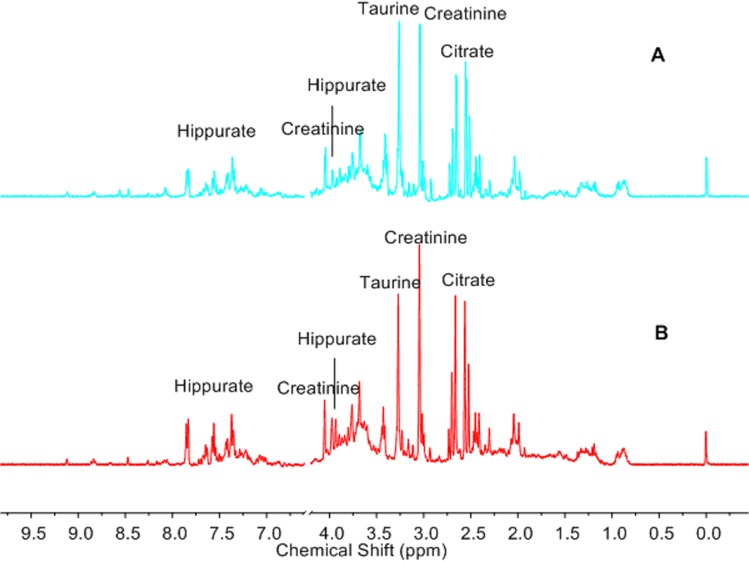
Assignment and intensity comparison of peaks with large loadings. (A) from the control group, (B) from the experimental group. The peaks with large loadings in the first three principal components (Fig. 3) are assigned in the ^1^H NMR spectra, and the corresponding metabolites and their changing trends in experimental group are shown in this figure. Compared with the control group, the urinary metabolites citrate, hippurate and creatinine are all increased in the experimental group, except for taurine, which decreased.

The peaks with large loadings in the first three PCs (Fig. 3) are assigned in the ^1^H NMR spectra (Fig. 4), and the corresponding metabolites and their changing trends in the experimental group are shown in Table 2.

**Table 2:** Metabolites with large loadings and their changing trends in the experimental group.

Principal component	Metabolites		Chemical shifts, δ^1^H (ppm)	Loadings	Changing trends
1	No. 1	Taurine	3.28* (t), 3.44 (t)	0.41	↓
	No. 2	Citrate	2.58* (d), 2.72 (d)	-0.36	↑
	No. 3	Creatinine	3.06* (s), 4.06 (s)	-0.29	↑
2	No. 1	Citrate	2.56* (d), 2.72 (d)	0.31	↑
	No. 2	Taurine	3.28* (t), 3.44 (t)	-0.27	↓
	No. 3	Hippurate	3.98* (d), 7.86 (d), 7.65 (t), 7.56 (t)	0.23	↑
3	No. 1	Taurine	3.44* (t), 3.28 (t)	0.34	↓
	No. 2	Hippurate	3.98 (d), 7.86* (d), 7.65 (t), 7.56 (t)	-0.26	↑

Note: s, singlet; d, doublet; t, triplet. The arrows indicate the increase (↑) or decrease (↓) in the levels of metabolites when compared with the controls.

* The loading of this peak was presented in the table to represent the loading of the assigned metabolite.

#### Meanings of PC 1

As shown in Table 2, the first representative metabolite of PC 1 is taurine, followed by citrate and by creatinine. Citrate is also the first representative metabolite of PC 2, which will be discussed later. The changes in taurine and in creatinine primarily reflect the metabolic stress reactions in organism due to chlorinated water.

Taurine, which is a derivative of cysteine, is called a conditionally essential amino acid and plays many protective roles in the body. Taurine acts as a substrate in the conjugation of bile acids; exerts osmoregulatory, membrane stabilizing, and cytoprotective effects; possesses antioxidative properties; modulates intracellular Ca^2+^ concentration; regulates neurotransmitters and ion movement; and reduces pro-inflammatory cytokines in various organs [17]. As an antioxidant, taurine can directly scavenge hypochlorous acid (HClO) and prevents changes in membrane permeability due to oxidative impairment [18]; as a protector of organs against toxicity, taurine reduces nitrosative stress and oxidative stress by increasing the activities of antioxidant enzymes and intracellular GSH and by normalizing the activities of Na^+^-K^+^-ATPase, Ca^2+^, and Mg^2+^-ATPase during various toxin-induced pathophysiological conditions [19–21].

Creatinine, which is a metabolic byproduct of skeletal muscle creatine and phosphocreatine, is generally eliminated from the kidneys by glomerular filtration with partial tubular excretion. Generally, urinary creatinine excretion is constant in healthy animals, and changes in urine excretion are usually connected with changes in muscle mass or in muscle energy metabolism. Considering that the body weights of the rats are so close between the two groups, the differences in muscle mass should be also slight. Therefore, the elevated level of urine creatinine detected in the EG rats might primarily be related to an unusual muscle energy metabolism.

#### Meanings of PC 3

Apart from taurine, which was discussed above, the second representative metabolites of PC 3 is hippurate (Table 1). Thus, PC 3 primarily reflects the changes in acid-base balance.

Hippurate belongs to the uremic toxin family and participates in various physiological processes of energy metabolism and of acid-base balance [22]. Hippurate is an inhibitor of glucose utilization in the muscle and in the kidneys, an inhibitor of glucose utilization in the kidneys and in the liver, a modulator of fatty acid metabolism, and a stimulator of ammoniagenesis. Metabolic acidosis stimulates hippurate synthesis in the liver and in the kidneys and increases urine excretion by the kidneys, whereas alkalinization decreases its synthesis and excretion [23]. The following possible mechanism of hippurate action in the correction of metabolic acidosis was proposed by Dzúrik et al. [22]: Metabolic acidosis stimulates hippurate synthesis and its tubular secretion; hippurate synthesized in the liver is released into the blood, from which hippurate is filtered in the glomeruli and taken up from the interstitium by the organic anion transport system; hippurate synthesized in the kidneys is secreted directly into primary urine; the increased hippurate promotes ammonia production by activating P-independent glutaminase (PIG) at the proximal luminal membrane, which initiates the metabolism of glutamine and of glutamate with ammonia formation, which is a dominant elimination product of H^+^.

#### Meanings of PC 2

As shown in Table 2, the first representative metabolite of PC 2 is citrate, followed by taurine and hippurate, which indicates that the elevated urine excretion of citrate might be related to the changes in taurine and hippurate.

Citrate is a dominant product of the tricarboxylic acid cycle (TCA) and occupies a critical crossroad step in the intermediary metabolism of most mammalian cells [24]. Citrate is synthesized in mitochondria and then becomes the entry substrate into the TCA. Its oxidation provides the major source of cellular ATP production; hence, elevated urinary excretion of citrate is an obvious sign of perturbed energy metabolism. Abnormal urinary excretion of citrate indicates mitochondrial inefficiencies in energy production and explains the biochemical basis of excessive fatigue and of weakness [25]. In addition to these roles, citrate also possesses a modulatory function in the correction of acid-base balance, as well as hippurate, because of its anion properties [25].

Thus, PC 2 primarily reflects the roles of citrate in energy metabolism and in acid-base balance. Considering the differences between the two groups are not significant on PC 2 (p>0.05), the modulatory functions of citrate in energy metabolism and in acid-base balance may be not enough to make significant differences.

### Metabolism of chlorination DBPs

**Fig 5:**
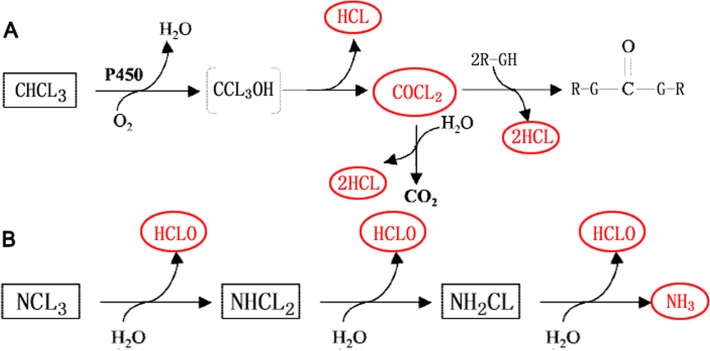
Metabolism of the representative chlorination disinfection by-products (DBPs) in swimming pools. (A) chloroform (CHCl_3_), (B) Chloramines. CHCl_3_ is primarily metabolized oxidatively to trichloromethanol and spontaneously decomposed to the electrophilic phosgene (COCl_2_). COCl_2_ is highly reactive, and binds covalently to cell components containing nucleophilic groups, and may be hydrolyzed by reacting with water, yielding CO_2_ and HCl. Chloramines, including NH_2_Cl, NHCl_2_ and NCl_3_, can change into one another easily while producing HClO and NH_3_. Overall, DBP-induced toxicity primarily originates from 4 reactive compounds, HClO, COCl_2_, HCl and NH_3_, which are marked with red in this figure.

Chlorine is a necessary element for our bodies, and nontoxic. HClO is the active ingredient of chlorination disinfectants, removing a variety of parasites, bacteria and viruses. However, although attacking microbes and viruses, HClO also reacts with many pool water substances and produces various DBPs. DBPs can be inhaled and ingested during swimming or absorbed dermally, and two classes of DBPs, chloroform and chloramines, have been the focus of most swimming pool studies thus far [26]. To better understand the corresponding metabolic stress pathways, the metabolism of these two classes of DBPs is illustrated in Fig. 5.

Chloroform is primarily metabolized in the liver, which explains its hepatoxicity; however, chloroform metabolism also occurs in other tissues, such as the kidneys. Chloroform metabolism may occur via two pathways, oxidative and reductive, but primarily via the oxidative pathway (Fig. 5A), except under special conditions of high chloroform doses in preinduced animals [27]. Extensive rodent studies have demonstrated that chloroform may be metabolized oxidatively to trichloromethanol and spontaneously decomposed to the electrophilic phosgene (COCl_2_) [28–29]. COCl_2_ is highly reactive, and binds covalently to cell components containing nucleophilic groups, including proteins, reduced glutathione, and phospholipid polar heads [30–31], and may be hydrolyzed by reacting with water, yielding carbon dioxide and hydrochloric acid (HCl). Chloramines, including monochloramine (NH_2_Cl), dichloramine (NHCl_2_) and trichloramine (NCl_3_), are not persistent and can change into one another easily while producing HClO and NH_3_ (Fig. 5B).

Overall, DBP-induced toxicity primarily originates from 4 reactive compounds, HCLO, COCL_2_, HCl and NH_3_ (Fig. 5). These compounds are all highly toxic to cells. These compounds can attack cells directly or indirectly by reacting with amino acids, destroying membranes, changing the construction and function of proteins and lipids, unbalancing the acid-base balance, blocking metabolism and by inducing respiratory burst.

### Corresponding metabolic stress pathways

**Fig 6:**
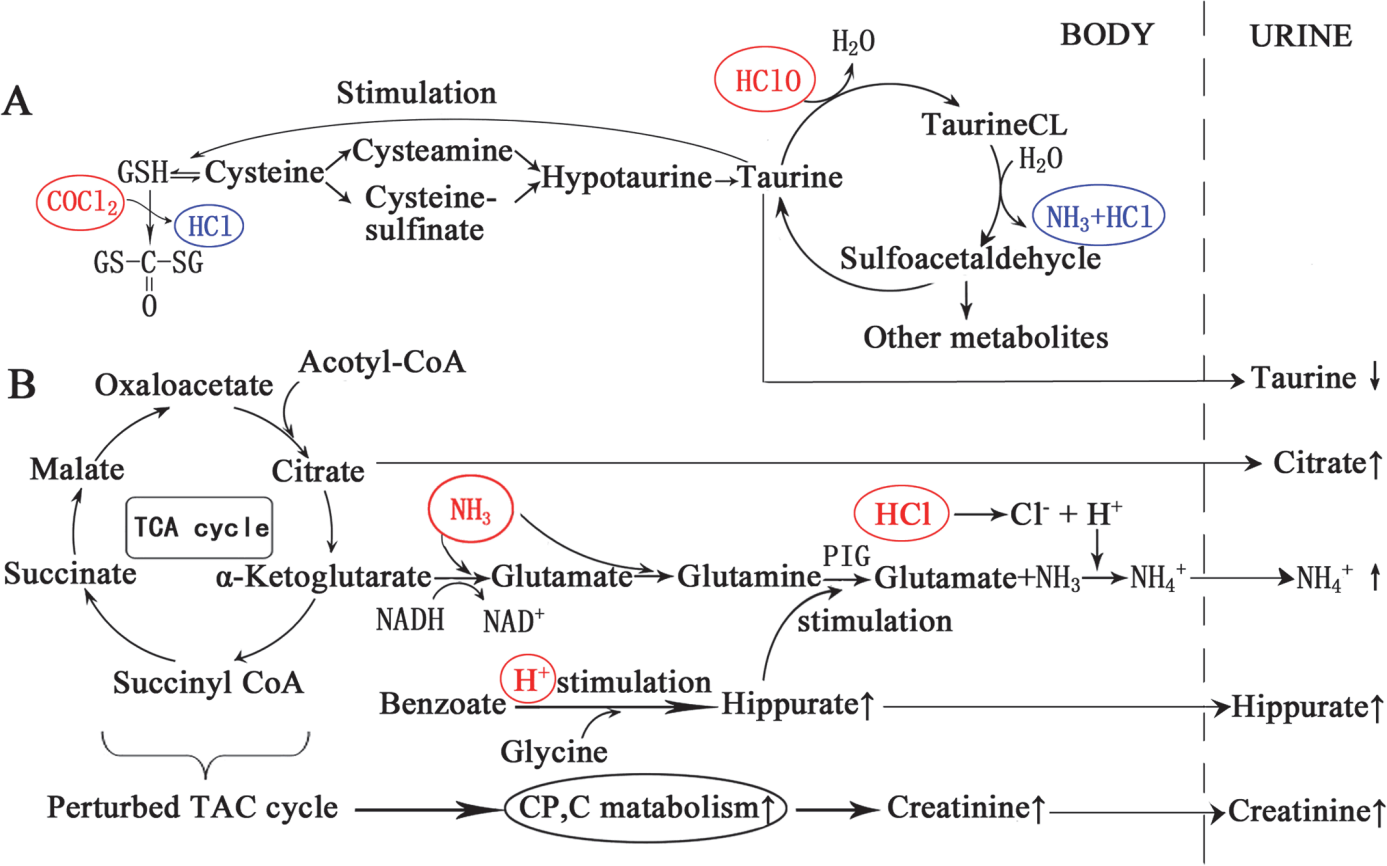
Corresponding metabolic stress pathways to the chlorination disinfection by-products (DBPs). (A) elimination of HClO and of COCl_2_, (B) elimination of NH_3_ and of HCl. First, taurine reacts directly with HClO to form taurine chloramines (TaurineCL). Second, by increasing GSH activities, taurine also plays an important role in eliminating COCl_2_. To convert TaurineCL to sulfoacetaldehyde and COCl_2_ to CO(GS)_2_, NH_3_ and HCl are formed. To eliminate NH_3_ and H^+^, hippurate and citrate play important roles. The increased H^+^ stimulates hippurate synthesis, then the increased hippurate stimulates the formation of ammonia in kidneys, and finally, ammonia and H^+^ are secreted into urine as NH_4_^+^, thus leading to an increased urinary excretion of hippurate and of citrate because of their anion properties.

To eliminate the health hazards of these 4 compounds, the corresponding metabolic stress reactions occur in organisms. As shown in Table 1, taurine is the No. 1 representative metabolite of PC 1, the No. 2 representative metabolite of PC 2 and the No. 1 representative metabolite of PC3, which indicates that taurine plays the key role in the stress reactions induced by DBPs. As shown in Fig. 6A, first, taurine can react directly with HClO to form taurine chloramines (TaurineCL) [32–33]. Second, by increasing the activities of intracellular GSH, taurine also plays an important role in eliminating COCl_2_. To convert TaurineCL to sulfoacetaldehyde and COCl_2_ to CO(GS)_2_, NH_3_ and HCl are formed in these procedures (Fig. 6A) [34], thus explaining why taurine is also involved in the acid-base balance and is related to the urinary excretion of hippurate and of citrate (this function will be discussed in the next section). Additionally, because taurine reacts directly with HClO and indirectly with other oxidative radicals, the urinary excretion of the EG rats showed a declining trend.

As above-mentioned, NH_3_ and HCl are also highly toxic to cells. To eliminate ammonia toxicity and acidosis induced by these two compounds, NH_3_ and H^+^ must be excreted as soon as possible. In these procedures, hippurate and citrate play important roles. First, the increased H^+^ stimulates hippurate synthesis in the liver and in the kidneys, thus increasing the hippurate plasma concentration. Then, the increased hippurate stimulates PIG desamidating glutamine with the formation of ammonia, and finally, ammonia and H^+^ are secreted into urine as NH_4_^+^ (Fig. 6B) [22]. To balance increased NH_4_^+^ excretion, the excretion of hippurate and citrate are also increased because of their anion properties.

However, to eliminate the ammonia toxicity and acidosis, energy metabolism in cells is seriously impaired because two substrates of the TCA, citrate and α-ketoglutarate, are involved in the excretion of NH_3_ and of H^+^ (Fig. 6B). On the one hand, elevated ammonia stimulates glutamate dehydrogenase to catalyze the reductive amination of α-ketoglutarate, resulting in the depletion of α-ketoglutarate and of NADH. On the other hand, to balance the increased NH_4_^+^ excretion, citrate, which is another substrate of the TCA, is secreted more than normal. Thus, the TCA and oxidative phosphorylation are inhibited [25]. To meet the increased demand of energy for recovery from the fatigue caused by swimming training and by stress reactions against the toxic compounds, the skeletal muscle must catalyze more phosphocreatine and creatine, thus resulting in elevated urine creatinine. Meanwhile, the inhibited TCA and the inhibited oxidative phosphorylation also explain the inferior performance of the EG rats in the swimming capacity test.

## Conclusions

In summary, the 12-week swimming training in chlorinated pool water did induce some disease symptoms, an impairment of training effects and a significant change in the metabolic profiles, although the growth and behaviors of the experimental animals were not affected. The bloody noses, bloody eyes, lackluster fur and hair removal reflected the direct irritation of the DBPs to the respiratory tract, eyes and skin because these organs were in direct contacted with the DBPs. However, possibly because of the protection from nasal mucous, the respiratory tract showed some adaptability to the DBPs. Thus, the eyes and skin might be the organs that require greater attention for permanent damage. Symptoms found in the liver and lung sections indicate that the liver is most likely the most possible target organ of DBPs, and training intensity, training frequency and water choking may be the primary factors for lung damage induced by swimming, instead of chlorination. The result of the swimming capacity test showed that training effects were significant affected by chlorinated water through perturbing the TCA cycle and oxidative phosphorylation. Through analyzing the changes in metabolic profiles using a multivariate analysis, corresponding metabolic stress pathways were proposed, as shown in Fig. 6, in which a defense system centering on taurine and related metabolites is presented. Based on these pathways, the underlying toxicological mechanisms of DBPs are illustrated, and corresponding countermeasures can be developed.

## Supporting Information

S1 ARRIVE Guidelines Checklist Completed “The ARRIVE Guidelines Checklist” for reporting animal data in this manuscript. (PDF)

S1 Table The normalized urine metabolite data of the individual animal (data < 0.000001 excluded). (XLS)
